# A candidate gene identification strategy utilizing mouse to human big-data mining: “3R-tenet” in COPD genetic research

**DOI:** 10.1186/s12931-018-0795-y

**Published:** 2018-06-06

**Authors:** Sangeetha Vishweswaraiah, Leema George, Natarajan Purushothaman, Koustav Ganguly

**Affiliations:** 1 SRM Research Institute, SRM University, Chennai, 603203 India; 2 Department of Genetic Engineering, School of Bioengineering, Faculty of Engineering and Technology, SRM University, Chennai, 603203 India; 3 Work Environment Toxicology, Institute of Environmental Medicine, Karolinska Institutet, Box 287, SE-171 77 Stockholm, Sweden

**Keywords:** 3R, Alternate models, COPD, Asthma, Lung, Gene, Transcriptomics

## Abstract

**Background:**

Early life impairments leading to lower lung function by adulthood are considered as risk factors for chronic obstructive pulmonary disease (COPD). Recently, we compared the lung transcriptomic profile between two mouse strains with extreme total lung capacities to identify plausible pulmonary function determining genes using microarray analysis (GSE80078). Advancement of high-throughput techniques like deep sequencing (eg. RNA-seq) and microarray have resulted in an explosion of genomic data in the online public repositories which however remains under-exploited. Strategic curation of publicly available genomic data with a mouse-human translational approach can effectively implement “3R- Tenet” by reducing screening experiments with animals and performing mechanistic studies using physiologically relevant in vitro model systems. Therefore, we sought to analyze the association of functional variations within human orthologs of mouse lung function candidate genes in a publicly available COPD lung RNA-seq data-set.

**Methods:**

Association of missense single nucleotide polymorphisms, insertions, deletions, and splice junction variants were analyzed for susceptibility to COPD using RNA-seq data of a Korean population (GSE57148). Expression of the associated genes were studied using the Gene Paint (mouse embryo) and Human Protein Atlas (normal adult human lung) databases. The genes were also assessed for replication of the associations and expression in COPD−/mouse cigarette smoke exposed lung tissues using other datasets.

**Results:**

Significant association (*p* <  0.05) of variations in 20 genes to higher COPD susceptibility have been detected within the investigated cohort. Association of *HJURP, MCRS1* and *TLR8* are novel in relation to COPD. The associated *ADAM19* and *KIT* loci have been reported earlier. The remaining 15 genes have also been previously associated to COPD. Differential transcript expression levels of the associated genes in COPD- and/ or mouse emphysematous lung tissues have been detected.

**Conclusion:**

Our findings suggest strategic mouse-human datamining approaches can identify novel COPD candidate genes using existing datasets in the online repositories. The candidates can be further evaluated for mechanistic role through in vitro studies using appropriate primary cells/cell lines. Functional studies can be limited to transgenic animal models of only well supported candidate genes. This approach will lead to a significant reduction of animal experimentation in respiratory research.

**Electronic supplementary material:**

The online version of this article (10.1186/s12931-018-0795-y) contains supplementary material, which is available to authorized users.

## Background

Progress in the genomics technologies continue to tremendously advance our understanding of chronic lung diseases like asthma, chronic obstructive pulmonary disease (COPD), and idiopathic pulmonary fibrosis. COPD alone is the 4th leading cause of death globally [http://www.who.int/mediacentre/factsheets/fs310/en/]. Genetic predisposition is considered to be an important risk factor for COPD susceptibility. This is evident from the fact that only 15–20% of smokers develop COPD [1, 2]. Thus, candidate gene identification has been a major focus for COPD research. This has also lead to the extensive use of inbred mouse strains for screening experiments and also to the development of transgenic mouse models to identify genetic susceptibility, elucidation of molecular patho-mechanisms and toxicity testing in COPD research. However, a spin-off of the popularity of transgenic strains to explore gene-function relationships is the increased animal usage [3]. Another corresponding concern is the large number of animals bred that are genetically unsuited for the experiment. Breeding surplus often counts for 50% of the offspring [3]. Moreover, the relevance of a mouse with a single gene inserted or knocked out for studying human diseases is also questioned. This is mainly because complex traits are multi-gene controlled that do not follow Mendelian pattern of inheritance. Pulmonary function and COPD are classic examples of such phenomenon [4–18]. Yet we believe, transgenic models may continue to serve as important resources for studying gene-function relationships particularly in the field of respiratory research. However, the strategy to select candidate genes for using transgenic models to study COPD and other chronic lung diseases is an important issue that warrants attention.

Practice of the “3R tenet”-replacement, reduction and refinement warrants a scientist to adequately evaluate non-animal alternatives prior to performing animal experiments [19, 20]. Strategic genomics data mining using the public repositories can put in practice the “3R-tenet” more effectively by: i) reducing screening experiments with animals, ii) performing mechanistic studies in physiologically relevant alternate in vitro model systems and using advanced technologies like RNAi or CRISPR-Cas9 for understanding gene-function relationships, and iii) performing in vivo functional testing using transgenic animal models limited to well supported candidate genes.

An accelerated decline in lung function is considered to be the earliest indicator for predisposition, onset and COPD severity assessment. We previously identified mouse strains (C3H/HeJ and JF1/MsJ) with extreme total lung capacities [5, 21, 22]. Recently, we performed a large-scale microarray study (GSE80078) to compare the lung transcript expression profiles of C3H/HeJ and JF1/MsJ mice at the completion of: (I) embryonic lung development; (II) bulk alveolar formation and (III) lung growth and maturity [18]. The generated microarray data provides a publicly available resource for performing genetic association studies as well as functional and mechanistic investigations to understand pulmonary function development and chronic lung disease (eg. COPD) susceptibility [18]. Lung developmental pathways are recollected in genetic subroutines during repair and remodeling processes following lung injury. Therefore, it is plausible that an individual with hindered lung development may have an inefficient repair/remodeling process thereby predisposing them to chronic lung diseases like COPD [23–25]. A study by Lange et al. [26] showed that forced expiratory volume in 1 s (FEV_1_) in early adulthood is important for the genesis of COPD and that accelerated decline in FEV_1_ is not an obligate feature of COPD. Therefore, in this work, we performed an *in-silico* study, testing the association of functional variations within human orthologs of mouse lung function candidate genes [18] in a publicly available RNAseq dataset of a COPD cohort [27].

### Methods

**Fig. 1:**
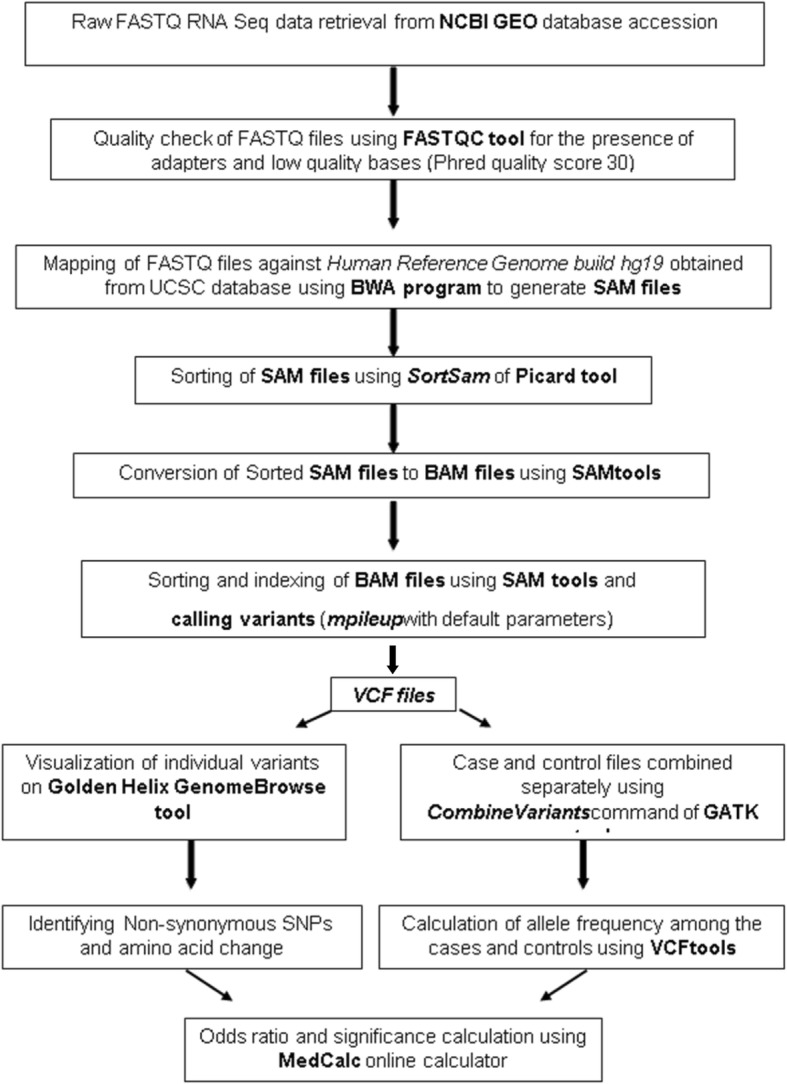
Strategic workflow to screen mouse lung developmental genes for their association within a human chronic obstructive pulmonary disease (COPD) cohort transcriptomic (RNAseq) data

Figure 1 illustrates the overall analysis strategy followed in this study. We focused on the missense single nucleotide polymorphisms (SNPs), insertions, deletions and splice site variations for detecting the functional relevance of the associations. Lung transcriptome data (RNA-seq; GSE57148) from a Korean cohort [27] were analyzed to call the variants and to identify the SNPs with significant (*p* <  0.05) allelic frequency differences between the COPD cases and controls.

### Selection of mouse genes

Mouse lung microarray dataset was retrieved (GSE80078) from our recently completed project contrasting C3H/HeJ (large total lung capacity) and JF1/MsJ (small total lung capacity) [18]. Genes exhibiting increased/decreased transcript expression levels by ≥2 fold in the lungs of JF1/MsJ mice compared to C3H/HeJ were selected for performing the association studies. We also included the top 20 genes identified in Kim et al. [27] study and other COPD associated genes by literature survey resulting in a total of 494 genes for screening. Human orthologs of some genes were not found and many were RIKEN or expressed sequence tags. Therefore, the final search list constituted of 355 genes (Additional file 1: Table S1).

### Human lung transcriptome data

A publicly available RNA-seq dataset from a Korean cohort consisting of 98 COPD cases and 91 control subjects was selected for the analysis [27]. Based on our search term [(COPD RNA seq human) and “*Homo sapiens*”] this was the largest available COPD RNA-seq dataset at the Gene expression Omnibus (GEO) database. The raw FASTQ files of paired end reads representing the transcriptome of control and cases were retrieved from the GEO database at the National Centre for Biological Information (NCBI) through accession number GSE57148 (http://www.ncbi.nlm.nih.gov/geo/query/acc.cgi?acc=GSE57148) [27].The quality of the raw FASTQ files were analyzed using FASTQC (http://www.bioinformatics.babraham.ac.uk/projects/fastqc/) for the presence of sequencing adapters and low-quality bases (Phred quality score 30). The quality filtered FASTQ files (Paired end) for each sample were then mapped against the Human Reference Genome build hg19 (http://hgdownload.soe.ucsc.edu/goldenPath/hg19/bigZips/chromFa.tar.gz)usingthe Burrows Wheeler alignment (BWA) tool version 0.7.10 (http://bio-bwa.sourceforge.net/). The whole genome alignment was performed using ‘*BWA-MEM’* algorithm with default parameters [28].

The aligned reads in the Sequence Alignment/Map (SAM) format were then sorted using ‘*SortSam’* algorithm of Picard tool v.1.118 (https://sourceforge.net/projects/picard/). The Sorted SAM file was converted to binary version of a SAM file (BAM file) using the SAMtools (http://samtools.sourceforge.net/). The resulting BAM file was then sorted and indexed using SAMtools (http://samtools.sourceforge.net/) for variant calling. The *‘mpileup’* algorithm of SAM tools was used for calling variants from the sorted BAM file using default parameters. The resulting variant calling file (VCF) containing SNPs was used for the further downstream analysis. The VCF files generated from COPD cases and controls were separately combined using *CombineVariants* command in Genome Analysis Tool Kit (GATK) v.2.3.9 (https://www.broadinstitute.org/gatk/). The allele frequency in cases and controls were calculated using VCF tools v.0.1.12a (http://vcftools.sourceforge.net/). The calculated allelic frequencies were considered to compare the differences in SNPs frequencies among the COPD cases and the controls.

### Statistics

The relative odds with the “cross-products” ratio was used for calculating statistical significance. Followed by odds ratio estimation, the confidence interval was calculated. Ninety five percent confidence level was considered for the estimation [29]. The odds ratio and the significance of the associations were calculated using a statistical tool MedCalc (https://www.medcalc.org/calc/odds_ratio.php). Single variant analysis was performed and the raw *p* <  0.05 was considered as significant.

### In silico assessment of functional consequence of the associated variations on protein biochemistry

The polymorphisms with the significant allelic frequency differences between the COPD cases and controls were further analyzed using the visualization tool ‘*Golden Helix GenomeBrowse’* (http://www.goldenhelix.com) to assess the plausible effect of SNPs on protein biochemistry or splicing events. *Prosite*’ tool of ExPASy [30] was used to analyze the effect of amino acid changes on the functional domains of proteins.

### In silico lung expression domain studies of associated genes

Transcript expression of the significantly associated genes were screened in embryonic mouse lungs using the online database “GenePaint” [31]. “The Human Protein Atlas” database [32] was used to identify the immuno-positive lung cells for the significantly associated genes in normal adult human lung.

### Lung transcript expression levels of the associated genes in COPD and cigarette smoke exposed mice

The associated 20 genes were scanned for differential transcript expression in several COPD and/ or emphysematous lung tissues (GSE: 29133, 22,148, 1650, 47,460 and 54,837) [33–37] as well as in mouse cigarette smoke exposed lungs (GSE: 8790, 7310, 17,737, and 76,205) [38–40] using microarray/RNA-seq datasets from GEO database.

## Results

A stringent cut off ratio of ≥2 fold increased/decreased was used to select the mouse lung function developmental genes (GSE80078) for association studies in the RNA-seq dataset of the investigated Korean COPD cohort (GSE57148). Our study identified significant association of 16 non-synonymous SNPs, 4 splice junction variations and 3 insertions involving 20 genes out of the 355 screened genes to higher COPD susceptibility in the investigated cohort (Table 1).

**Table 1:** Details of the gene and corresponding single nucleotide polymorphism (SNP) associated to chronic obstructive pulmonary disease (COPD) susceptibility

Gene	Gene name	Entrez ID	Associated SNP	Chromosomal Location	Ref allele/Alt allele	Ref AA/Alt AA	Odds ratio	95% CI	z-statistic	Significance level	Association to COPD
*ABCA10*	ATP-binding cassette, sub-family A, member 10	10,349	rs4968849	17: 67178316	A/G	M/T	4.09	1.11 to 15.01	2.125	0.0336	Novel loci, gene associated previously (NLGAP)
*ADAM19*	ADAM metallopeptidase domain 19	8728	rs1422795	5: 156936364	T/C	S/G	6.21	2.26 to 17.00	3.555	0.0004	Loci and gene associated previously
*BHLHE41*	Basic helix-loop-helix family, member e41	79365	rs11048413	12: 26275555	G/A	A/V	5.29	2.71 to 10.35	4.879	< 0.0001	NLGAP
CD200	CD200 molecule	4345	rs1131199	3: 112059768	C/G	S/C	2.3773	1.11 to 5.05	2.248	0.0246	NLGAP
*CYBB*	Cytochrome b-245, beta polypeptide	1536	Novel	X: 37658269	C/A	Q/K	2.9091	1.35 to 6.26	2.726	0.0064	NLGAP
*GATM*	Glycine amidinotransferase	2628	rs1288775	15: 45661678	T/A	Q/H	2.4309	1.35 to 4.36	2.974	0.0029	NLGAP
*GBP1*	Guanylate binding protein 1, interferon-inducible	2633	rs1048425	1: 89522646	G/C	T/S	3.2611	1.70 to 6.24	3.566	0.0004	NLGAP
*HJURP*	Holliday junction recognition protein	55355	rs2286430	2: 234761225	C/T	E/K	3.36	1.42 to 7.94	2.768	0.0056	Novel
*KIT*	V-Kit, sarcoma viral oncogene homolog	3815	rs3822214	4: 55593464	A/C	M/L	5.05	1.07 to 23.74	2.054	0.04	Loci and gene associated previously
*LEPR*	Leptin receptor	3953	rs1137101	1: 66058513	A/G	Q/R	10.39	5.00 to 21.58	6.28	< 0.0001	NLGAP
*LMO7*	LIM domain 7	4008	Insertion	13: 76383319	A to G Insertion	–	3.6316	1.99 to 6.62	4.209	< 0.0001	Novel insertion, gene associated Previously (NIGAP)
*LMO7*	LIM domain 7	4008	Insertion	13: 76429504	T insertion	–	3.5531	1.50 to 8.36	2.903	0.0037	NIGAP
*LRP1*	Low density lipoprotein receptor-related protein 1	4035	Splice junction	12: 57605134	G/C	–	10.22	1.28 to 81.58	2.195	0.0282	Novel splice site; gene associated Previously (NSSGAP)
*MCRS1*	Microspherule protein 1	10445	Splice junction	12: 49957330	C/T	–	3.365	1.57 to 7.19	3.127	0.0018	Novel
*POP4*	Processing of precursor 4, ribonuclease P/MRP subunit (*S. cerevisiae*)	10775	Splice junction	19: 30101540	G/A	–	2.7669	1.31 to 5.83	2.673	0.0075	NSSGAP
*PTCH1*	Patched 1	5727	Splice junction	9: 98242373	G/T	–	11.41	2.58 to 50.37	3.213	0.0013	NSSGAP
*SCN7A*	Sodium channel, voltage-gated, type VII, alpha subunit	6332	rs7565062	2: 167334085	G/T	T/N	4.4175	1.97 to 9.90	3.606	0.0003	NLGAP
*SCN7A*	Sodium channel, voltage-gated, type VII, alpha subunit	6332	Insertion	2: 167289263	AG Insertion	–	3.3561	1.17 to 9.57	2.263	0.0237	NIGAP
*SCN7A*	Sodium channel, voltage-gated, type VII, alpha subunit	6332	rs6738031	2: 167279922	C/A	M/I	2.2817	1.09 to 4.76	2.198	0.028	NLGAP
*SLFN12L*	Schlafen family member 12 like	100,506,736	rs2304968	17: 33805150	T/C	Y/C	2.2	1.07 to 4.51	2.151	0.0315	NLGAP
*TLR8*	Toll-like receptor 8	51,311	rs3764880	X: 12924826	A/G	M/V	2.97	1.11 to 7.91	2.181	0.0292	Novel
*TTC5*	Tetratricopeptide repeat domain 5	91875	rs3742945	14: 20770036	T/C	Q/R	2.1591	1.14 to 4.07	2.375	0.0176	NLGAP
*VEPH1*	Ventricular zone expressed PH domain homolog 1 (zebrafish)	79674	rs11918974	3: 157081324	A/G	S/P	1.8668	1.04 to 3.32	2.116	0.0343	NLGAP

*AA* amino acids, *rs* reference sequence, *Ref* reference, *Alt* altered, *CI* confidence interval

*p* < 0.05 was considered as significant

### Association of novel and previously reported genes to COPD

The 20 associated genes include: ATP binding cassette subfamily A member 10 (*ABCA10*); a disintegrin and metallopeptidase domain 19 (*ADAM19*); basic helix-loop-helix family member e41 (*BHLHE41),* CD200 molecule (*CD200*); cytochrome b-245, beta polypeptide (*CYBB*); glycine amidinotransferasec (*GATM);* guanylate binding protein 1 (*GBP1*); holliday junction recognition protein (*HJURP*); KIT proto-oncogene receptor tyrosine kinase (KIT); leptin receptor (*LEPR*); LIM domain 7 (*LMO7*); LDL receptor related protein 1 (*LRP1*); microspherule protein 1 (*MCRS1*); processing of precursor 4, ribonuclease P/MRP subunit (*POP4*); Patched 1 (*PTCH1*); sodium channel, voltage-gated, type VII, alpha subunit (*SCN7A*); schlafen family member 12 like (*SLFN12L*); toll like receptor 8 (*TLR8*); tetratricopeptide repeat domain 5 (*TTC5*) and ventricular zone expressed PH domain homolog 1 (*VEPH1*).

Our analysis, identified *HJURP* (rs2286430), *MCRS1* (splice junction), and *TLR8* (rs3764880) as three novel COPD associated genes (Table 1). The variations (missense SNPs/splice junction variations) on *ABCA10* (rs496849), *BHLHE41* (rs11048413), *CD200* (rs1131199), *CYBB* (not reported in dbSNP), *GATM* (rs1288775), *GBP1* (rs1048425), *LEPR* (rs1137101), *LMO7* (2 insertions), *LRP1* (splice junction), *POP4* (splice junction), *PTCH1* (splice junction), *SCN7A* (rs7565062, rs6738031, 1 insertion), *SLFN12L* (rs2304968), *TTC5* (rs3742945), and *VEPH1* (rs11918974) are located on genes previously associated to COPD (Table 1). The associated SNPs on *ADAM19* (rs1422795) and *KIT* (rs3822214) have been previously reported in relation to COPD (Table 1).

### In silico protein domain and gene/protein expression analysis

In silico protein domain analysis revealed the *ADAM19* (rs1422795) variation at the position of Chr5: T-156936364-C resulting in an amino acid exchange of Ser17Gly (polar to non-polar) to be located within the ADAM metalloprotease domain (Additional file 1: Figure S1). None of the other amino acid changes were located within functional domains of the proteins. In silico transcript expression domain analysis using the Gene Paint database (Additional file 1: Table S2) revealed detectable lung expression of *Adam19, Cd200, Cybb, Mfleg* (*HJURP)*, *Kit, Lepr, Lmo7, Lrp1, Mcrs1, Pop4* and *Ptch1* in mouse embryo (E14.5; at pseudoglandular stage of lung development). This further attests the role of the mentioned 11 genes in the process of lung development. Impairment in the regulation and functionality of lung developmental genes may result in predisposition to chronic lung diseases like COPD. In silico lung protein expression domain analysis using the Human Protein Atlas revealed detectable immuno-expression of 18 associated genes in macrophages and/or pneumocytes and/or nasopharynx (respiratory epithelial cells) and/or bronchus (respiratory epithelial cells) (Additional file 1: Table S2). Immuno-expression of BHLHE41 and GATM were not detectable in the normal human lung tissue. Detection of expression of the significantly associated COPD susceptibility genes within specific cell types of the normal human lung further supports their specific role in the normal lung physiology. Additional file 1: Figures S1-S4 shows the expression of HJURP, MCRS1 and TLR8 in mouse embryonic lungs and normal adult human lungs. However, human protein atlas does not provide information on the expression of proteins in COPD tissues. Therefore, we investigated the transcript expression levels of the associated genes using available datasets on the lungs of COPD patients and mouse exposed to cigarette smoke.

The associated SNP rs2286430 (C/T) located on HJURP results in an amino acid change of glutamic acid (Glu: acidic, polar and negatively charged) to lysine (Lys: basic, polar and positively charged) in HJURP. Low to medium intensity of HJURP immune positive macrophages, pneumocytes, respiratory epithelial cells have been demonstrated in normal human lung tissue (Additional file 1: Figure S2) (Human Protein Atlas). *Hjurp* transcripts has been detected in mouse embryonic lungs (Additional file 1: Figure S2). *Mcrs1* is expressed in the mouse embryonic lungs (Additional file 1: Figure S3) (Gene Paint). Medium to high intensity immune-positive MCRS1 macrophages, pneumocytes, respiratory epithelial cells have been demonstrated in normal human lung tissue (Additional file 1: Figure S3) (Human Protein Atlas). TLR8 immuno-positive (high intensity) macrophages are reported in normal human lung (Additional file 1: Figure S4). The intensity of TLR8 immuno-positive staining in the respiratory epithelial cells is low (Additional file 1: Figure S4) whereas in pneumocytes and embryonic mouse lung TLR8/*Tlr8* was not detectable (Human Protein Atlas; Gene Paint).

### Lung transcript expression of the associated genes in other COPD cohorts and mouse studies

We investigated the transcript expression levels of the associated 20 genes in several COPD and/ or emphysematous lung tissue data sets. *SLFN12L* is the only gene not exhibiting any differential expression in any of the investigated datasets. A summary of the expression pattern of the 20 genes in the investigated COPD lung tissue datasets (GSE: 29133, 22,148, 1650, 47,460 and 54,837) is provided in Additional file 1: Table S3. Mouse cigarette smoke exposure experiments are also another valuable resource to evaluate molecular patho-mechanisms as tobacco smoking is the major risk factor for COPD. We therefore also evaluated the expression of the 20 associated genes in the datasets generated from lungs of mice exposed to cigarette smoke (GSE: 8790, 7310, 17,737, and 76,205) (Additional file 1: Table S4). In case of mouse studies, *Gbp1, Mcrs1, Ptch1, Slfn12l, and Ttc5* were the genes not exhibiting altered expression following cigarette smoke exposure. A summary of the expression pattern of the 20 genes in the cigarette smoke exposed mouse lung tissue datasets are provided in the Additional file 1: Table S4. Amongst the 20 candidate COPD genes identified in our study, transcripts of all except GBP1, MCRS1, PTCH1, SLFN12L and TTC5 are differentially expressed in both mouse cigarette smoke exposed lungs and human COPD/emphysematous lungs within the investigated datasets.

## Discussion

All datasets investigated in this study originated from the lung samples of human and mouse thereby confirming the tissue specificity (18, 27, 37–40). The dataset GSE57148 from Kim et al. (27) study consisting of 98 COPD patients and 91 control subjects from a Korean population. This was the largest available lung RNA-seq dataset of a COPD cohort in GEO database at the time of study. However, for association studies this is a small sample size. It is important to note that most of the association studies on COPD genetics and genomics of pulmonary function originates from populations with European ancestry. Therefore, the effect of ethnicity on the current findings cannot be ruled out. Additional file 1: Table S5 shows the difference in minor allele frequencies of the associated SNPs between Korean population (http://152.99.75.168/KRGDB/browser/mainBrowser.jsp) and global population (https://www.ncbi.nlm.nih.gov/SNP/) justifying the plausible differences in ethnicity.

Apart from lung specific expression of the associated genes, another strength of our study is the focus on missense SNPs (amino acid change), insertions, deletions, and splice junction variations thereby increasing the functional relevance of these associations. A genome-wide analysis of alternative splicing indicated that 40–60% of human genes undergo alternative splicing, often in a tissue specific manner [41–44]. On the other hand, since we performed the study using RNAseq data, our investigation is limited only to the exonic sequences and therefore could not detect any alterations within the promoter or intronic region. RNAseq data provides information only of a single strand. Thus, our study lacks information on the homozygosity of the identified associations. Availability of the genomic sequence of the same individuals would have overcome this drawback.

We detected association of 20 genes to higher susceptibility for COPD. Our findings on the association of SNPs located on *ADAM19 (rs1422795)* and *KIT* (rs3822214) to higher COPD susceptibility replicate the previous findings by other investigators [12, 45–48]. The rs11048413 SNP on *BHLHE41* causing an Ala298Val change have been associated to patient survival in lung adenocarcinoma. The Ala/Val or Val/Val genotype was associated to poor survival rate compared to Ala/Ala genotype [49]. The associated SNP on *GATM* (rs1288775) has been linked to lung cancer phenotypes with and without emphysema among African-American population but not among white Americans [50]. The SNP rs3764880 on *TLR8* has been associated to tuberculosis. The SNP rs3761624 also located on *TLR8* which has been associated to allergic rhinites in a Swedish population is in perfect linkage disequibrium with rs3764880 suggesting their complementary relationship [51].

The genes *ABCA10, BHLHE41, CD200, CYBB, GATM, GBP1, LEPR, LMO7, LRP1, POP4, PTCH1, SCN7A, SLFN12L, TTC5,* and *VEPH1* have been previously associated to COPD [52–68]. Moreover, we detected altered transcript expression of *ABCA10, ADAM19, BHLHE41, CD200, CYBB, GATM, GBP1, HJURP, KIT, LEPR, LMO7, LRP1, MCRS1, POP4, PTCH1, SCN7A, TLR8, TTC5 and VEPH1* in COPD and emphysematous lungs compared to control subjects in various datasets (GSE: 29133, 22,148, 1650, 47,460 and 54,837; Additional file 1: Table S3) [33–37]*.* In case of mouse lungs exposed to cigarette smoke, altered transcript expression was detected among *Abca8a (ABCA10), Adam19, Bhlhe41, Cd200, Cybb, Gatm, Hjurp, Kit, Lepr, Lmo7, Lrp1, Pop4, Scn7a, Tlr8, and Veph1* (GSE: 8790, 7310, 17,737, and 76,205; Additional file 1: Table S4) [38–40]. Effect of cigarette smoke exposure on COPD development may act as a confounding factor in the analysis of candidate susceptibility genes in this study. However, considering the concept of recapitulation of developmental pathways as genetic subroutines during lung repair/remodeling processes, altered regulation of the associated genes in both COPD-and cigarette smoke exposed mouse lungs seems to be reasonable. SNPs on *ADAM19* (rs2277027), *PTCH1* (rs16909898), *LRP1* (rs11172113) and hedgehog interacting protein (*HHIP;* rs12504628, rs1980057) have been associated to FEV_1_/forced vital capacity (FVC) ratio in samples of European ancestry [10, 12]. We previously reported decreased lung *Hhip* transcript levels in a mouse model lacking secreted phosphoprotein 1 (*Spp1*) with lower total lung capacity and enlarged alveolar size compared to control [8].

Based on the hypothesis on the origin of chronic lung diseases like COPD during the early life events [60–70], we could detect three novel (*HJURP, MCRS1 and TLR8*) COPD candidate genes and replicate the findings in 17 other studies using a mouse-human translational datamining approach. Gene set enrichment analysis [71] of the 20 associated genes identified COPD as one of the top enriched diseases (Additional file 1: Figure S5). HJURP is a centromeric protein (chaperone) that plays a central role in the incorporation and maintenance of histone H3-like variant CENPA at centromeres [72–74]. *MCRS1* have been implicated in epithelial-mesenchymal transition, metastasis and growth of lung cancer cells [75–77]. TLR8 is also expressed in human monocytes and myeloid dendritic cells and Th1-type immune response cells. Mucus hypersecretion is induced by dual TLR7/8 agonist [78, 79]. Similarly, the murine TLR8 is involved in the activation of innate immune responses [80]. Stimulation of TLR8 causes relaxation of airway smooth muscles thereby preventing broncho-constriction [81]. Association of *TLR8* have been also reported for pulmonary tuberculosis [82, 83], asthma and related atopic disorders [84].

## Conclusions

Through this study we could demonstrate a candidate gene identification strategy for COPD using mouse-human translational approach using existing genomic datasets in the public repositories. The strategy warrants validation in larger sample size and in multiple cohorts. Cigarette smoke exposure studies in mice are routinely practiced to model emphysema development, a commonly associated COPD phenotype, as it causes increased pulmonary inflammation, protease activity, oxidative stress and apoptosis [85]. However, cigarette smoke exposure in mice does not result in excessive mucus production or mucus cell metaplasia that is characteristic of COPD pathogenesis [85]. It is plausible that the different response to cigarette smoke exposure in human and mouse lungs may be due to their structural differences [85]. The inbred mouse strains also differ significantly in their resistance or susceptibility to emphysema development following cigarette smoke exposure as measured by airspace enlargement [86]. This variable susceptibility among inbred mouse strains to emphysematous change following cigarette smoke exposure may be attributed to their genetic constitution and differences in lung development. Most of the COPD transcriptomic profiling studies have been performed using lung tissue from severely diseased patients requiring lobectomy. On the contrary, COPD pathogenesis occurs over decades. Molecular mechanisms that are active during initial phase of the pathogenesis may be completely different compared to the end stage of the disease. Therefore, creation of a translational profile between mouse and human COPD transcriptomic data is challenging. In this respect, we share similar views as other investigators that it is important to carefully evaluate the common lung-biology and -pathobiology existing between mice and human prior to considering cigarette smoke exposure experiments in mouse models [85]. Single gene driven spontaneous emphysema developing mouse models [47] identified through physiological phenotyping (eg. pulmonary function screening) may serve an important tool to understand molecular patho-mechanism but this requires exhaustive supportive evidence prior to testing the transgenic model. One way of accumulating convincing supportive evidence is explained in the present work. Mechanistic studies to elucidate the role of the novel candidate genes can be performed using appropriate cell lines, primary cells and physiologically relevant in vitro models [87]. This approach would lead to a significant reduction of animal screening experiments in respiratory research.

## Additional file

Additional file 1: **Table S1.** List of the genes screened for association to higher Chronic Obstructive Pulmonary Disease (COPD) susceptibility. **Table S2.** Summary of the transcript (Gene Paint; mouse embryo) and protein (Human Protein Atlas) expression domains of the significantly associated chronic obstructive pulmonary disease (COPD) genes. **Table S3.** Analysis of lung transcript expression of the associated 20 genes in chronic obstructive pulmonary disease (COPD) and/ or emphysematous lung tissues using available datasets [GSE: 29133, 22,148, 1650, 47,460 and 54,837] in Genome Expression Omnibus (GEO) database. ↓: Decreased ↑: Increased ✓: significantly altered. **Table S4.** Analysis of l transcript expression of the associated 20 genes in mouse cigarette smoke exposed lungs using available datasets [GSE: 8790, 7310, 17,737, and 6205] in Genome Expression Omnibus (GEO) database. ↓: Decreased ↑: Increased ✓: significantly altered. **Table S5.** The difference in minor allele frequencies of the associated single nucleotide polymorphisms (SNPs) between Korean population and global population indicates the influence of ethnicity on the findings. The Korean population data was accessed from the KoreanDB: http://152.99.75.168/KRGDB/menuPages/firstInfo.jsp and http://152.99.75.168/KRGDB/browser/mainBrowser.jsp Global SNP data(dbSNP database): https://www.ncbi.nlm.nih.gov/SNP/. **Figure S1.** Analysis of protein domain and functional sites in the “A Disintegrin and metallopeptidase domain 19” (ADAM19). **Figure S2.** Transcript (Gene Paint; mouse embryo) and protein expression (Human Protein atlas; normal lung) domain of holliday junction recognition protein (*HJURP*). **Figure S3.** Transcript (Gene Paint; mouse embryo) and protein expression (Human Protein atlas; normal lung) domain of microspherule protein 1 (MCRS1). **Figure S4.** Protein expression (Human Protein atlas; normal lung) domain of toll like receptor 8 (TLR8). **Figure S5.** Gene-set enrichment analysis for the associated 20 genes for (A) cellular component enrichment (B) biological process enrichment (C) molecular function enrichment (D) diseases enrichment using Enrichr interactive enrichment analysis tool [71]. (PDF 1296 kb)

## Abbreviations

BAM: Binary version of a SAM file
BWA: Burrows Wheeler Alignment
GATK: Genome Analysis Tool Kit
GEO: Gene Expression Omnibus
NCBI: National Center for Biotechnology Information
SAM: Sequence Alignment/Map format
UCSC: University of California, Santa Cruz
VCF: Variant Call Format
