# Supplementary material for: A candidate gene identification strategy utilizing mouse to human big-data mining: “3R-tenet” in COPD genetic research
Source: Respir Res. 2018 Jun 6;19:92. doi: 10.1186/s12931-018-0795-y (PMC5989378; doi:10.1186/s12931-018-0795-y)

Supplementary material

A candidate gene identification strategy utilizing mouse to human big-data mining: "3R-Tenet" in COPD genetic research

Sangeetha Vishweswaraiah[1], Leema George[1], Natarajan Purushothaman[2], Koustav Ganguly[1,3]

[1]SRM Research Institute, SRM University, Chennai 603203 India

[2]Department of Genetic Engineering; School of Bioengineering, Faculty of Engineering and Technology; SRM University, Chennai 603203 India

[3]Work Environment Toxicology; Institute of Environmental Medicine, Karolinska Institutet; Box 287, SE-171 77 Stockholm, Sweden

Sangeetha Vishweswaraiah	sangeethav.srm@gmail.com
Leema George	leemag.srm@gmail.com
Natarajan Purushothaman	purushothaman.n@ktr.srmuniv.ac.in
Koustav Ganguly	koustav.ganguly@ki.se

Correspondence: Koustav Ganguly, PhD

Unit of Work Environment Toxicology, Institute of Environmental Medicine, Karolinska Institutet; Box 287, SE-171 77 Stockholm, Sweden

Email: koustav.ganguly@ki.se; Phone: +46-0852487133

This study was supported by Department of Biotechnology, Government of India: BT/PR12987/INF/22/205/2015, VINNOVA (2016-01951) (K.G)

Supplementary table ST1: List of the genes screened for association to higher Chronic Obstructive Pulmonary Disease (COPD) susceptibility

Sl. No.	Gene Symbol	Gene Name	Entrez ID
1	ABCA10	ATP-binding cassette, sub-family A (ABC1), member 10	10349
2	ABCG1	ATP-binding cassette, sub-family G (WHITE), member 1	9619
3	ACSM1	Acyl-CoA synthetase medium-chain family member 1	116285
4	ADAM19	ADAM metallopeptidase domain 19	8728
5	ADAMTS15	ADAM metallopeptidase with thrombospondin type 1 motif, 15	170689
6	ADH1C	Alcohol dehydrogenase 1C (class I), gamma polypeptide	126
7	AFP	Alpha-fetoprotein	174
8	AGER	Advanced glycosylation end product-specific receptor	177
9	AKAP5	A kinase (PRKA) anchor protein 5	9495
10	AKR1E2	Aldo-keto reductase family 1, member E2	83592
11	ALAD	Aminolevulinate dehydratase	210
12	ALAS2	Aminolevulinate, delta-, synthase 2	212
13	ALB	Albumin	213
14	ALOX15	Arachidonate 15-lipoxygenase	246
15	ANGPTL7	Angiopoietin-like 7	10218
16	ANKRD1	Ankyrin repeat domain 1 (cardiac muscle)	27063
17	ANKRD63	Ankyrin repeat domain 63	100131244
18	APOL3	Apolipoprotein L, 3	80833
19	ARHGAP20	Rho GTPase activating protein 20	57569
20	ARMC2	Armadillo repeat containing 2	84071
21	ARMC3	Armadillo repeat containing 3	219681
22	ARNTL	Aryl hydrocarbon receptor nuclear translocator-like	406
23	ARSI	Arylsulfatase family, member I	340075
24	ASPN	Asporin	54829
25	ATP6V0D2	ATPase, H+ transporting, lysosomal 38kDa, V0 subunit d2	245972
26	BAIAP2L1	BAI1-associated protein 2-like 1	55971
27	BHLHE40	Basic helix-loop-helix family, member e40	8553
28	BHLHE41	Basic helix-loop-helix family, member e41	79365
29	BMP3	Bone morphogenetic protein 3	651
30	BPIFA1	BPI fold containing family A, member 1	51297
31	C10orf11	Chromosome 10 open reading frame 11	83938
32	C12orf57	Chromosome 12 open reading frame 57	113246
33	C16orf89	Chromosome 16 open reading frame 89	146556
34	C1S	Complement component 1, s subcomponent	716
35	C5	Complement component 5	727
36	CALML3	Calmodulin-like 3	810
37	CASC4	Cancer susceptibility candidate 4	113201
38	CASP12	Caspase 12 (gene/pseudogene)	100506742
39	CCDC141	Coiled-coil domain containing 141	285025

40	CCDC190	Coiled-Coil Domain Containing 190	339512
41	CCDC38	Coiled-coil domain containing 38	120935
42	CCDC59	Coiled-coil domain containing 59	29080
43	CCL11	Chemokine (C-C motif) ligand 11	6356
44	CCL15	Chemokine (C-C motif) ligand 15	6359
45	CCL16	Chemokine (C-C motif) ligand 16	6360
46	CCL17	Chemokine (C-C motif) ligand 17	6361
47	CCL18	Chemokine (C-C motif) ligand 18	6362
48	CCL22	Chemokine (C-C motif) ligand 22	6367
49	CCL23	Chemokine (C-C motif) ligand 23	6368
50	CCL3	Chemokine (C-C motif) ligand 3	6348
51	CCL5	Chemokine (C-C motif) ligand 5	6352
52	CCR5	Chemokine (C-C motif) receptor 5	1234
53	CCR8	Chemokine (C-C motif) receptor 8	1237
54	CD14	CD14 molecule	929
55	CD200	CD200 molecule	4345
56	CD200R1	CD200 receptor 1	131450
57	CD209	CD209 molecule	30835
58	CD36	CD36 molecule (thrombospondin receptor)	948
59	CD68	CD68 molecule	968
60	CD74	CD74 molecule, major histocompatibility complex, class II invariant chain	972
61	CD86	CD86 molecule	942
62	CDC123	Cell division cycle 123 homolog (S. cerevisiae)	8872
63	CFD	Complement factor D (adipsin)	1675
64	CFDP1	Craniofacial development protein 1	10428
65	CH25H	Cholesterol 25-hydroxylase	9023
66	CHI3L1	Chitinase 3-like 1 (cartilage glycoprotein-39)	1116
67	CHIA	Chitinase, acidic	27159
68	CHIA	Chitinase, acidic	27159
69	CHRDL1	Chordin-like 1	91851
70	CHRM3	Cholinergic receptor, muscarinic 3	1131
71	CHSY3	Chondroitin sulfate synthase 3	337876
72	CKLF	Chemokine-like factor	51192
73	CLCA3P	Chloride channel accessory 3, pseudogene	9629
74	CLDN1	Claudin 1	9076
75	CLEC2D	C-type lectin domain family 2, member D	29121
76	CLEC6A	C-type lectin domain family 6, member A	93978
77	CLEC7A	C-type lectin domain family 7, member A	64581
78	CLEC9A	C-type lectin domain family 9, member A	283420
79	CLIC5	Chloride intracellular channel 5	53405
80	COL28A1	Collagen, type XXVIII, alpha 1	340267
81	COPZ2	Coatomer protein complex, subunit zeta 2	51226

82	COX6A1	Cytochrome c oxidase subunit VIa polypeptide 1	1337
83	CP	Ceruloplasmin (ferroxidase)	1356
84	CPEB1	Cytoplasmic polyadenylation element binding protein 1	64506
85	CRY2	Cryptochrome 2 (photolyase-like)	1408
86	CTSE	Cathepsin E	1510
87	CTSH	Cathepsin H	1512
88	CTSK	Cathepsin K	1513
89	CTSS	Cathepsin S	1520
90	CXCL11	Chemokine (C-X-C motif) ligand 11	6373
91	CXCL12	Chemokine (C-X-C motif) ligand 12	6387
92	CXCL13	Chemokine (C-X-C motif) ligand 13	10563
93	CXCL8	C-X-C motif chemokine ligand 8	3576
94	CXCL8/ IL8	C-X-C motif chemokine ligand 8	3576
95	CXCR6	Chemokine (C-X-C motif) receptor 6	10663
96	CYBB	Cytochrome b-245, beta polypeptide	1536
97	CYP2E1	Cytochrome P450, family 2, subfamily E, polypeptide 1	1571
98	CYP2J2	Cytochrome P450, family 2, subfamily J, polypeptide 2	1573
99	DBP	D site of albumin promoter (albumin D-box) binding protein	1628
100	DCDC2B	Doublecortin domain containing 2B	149069
101	DEFB4A	Defensin, beta 4A	1673
102	DNASE1L3	Deoxyribonuclease I-like 3	1776
103	DPEP1	Dipeptidase 1 (renal)	1800
104	DRAM1	DNA-damage regulated autophagy modulator 1	55332
105	EGFLAM	EGF-like, fibronectin type III and laminin G domains	133584
106	EMCN	Endomucin	51705
107	EMR1	EGF-like module containing, mucin-like, hormone receptor-like 1	2015
108	ENTPD4	Ectonucleoside triphosphate diphosphohydrolase 4	9583
109	EPHA7	EPH receptor A7	2045
110	F7	Coagulation factor VII (serum prothrombin conversion accelerator)	2155
111	FABP1	Fatty acid binding protein 1, liver	2168
112	FAM13A	Family with sequence similarity 13, member A	10144
113	FAM198B	Family with sequence similarity 198, member B	51313
114	FAR2	Fatty acyl coa reductase 2	55711
115	FCER2	Fc fragment of ige, low affinity II, receptor for (CD23)	2208
116	FCGR1A	Fc fragment of igg, high affinity Ia, receptor (CD64)	2209
117	FETUB	Fetuin B	26998
118	FGF1	Fibroblast growth factor 1 (acidic)	2246
119	FGG	Fibrinogen gamma chain	2266
120	FIRRE	Firre intergenic repeating RNA element	286467
121	FMO1	Flavin containing monooxygenase 1	2326
122	FPR1	Formyl peptide receptor 1	2357

123	FST	Follistatin	10468
124	FZD6	Frizzled family receptor 6	8323
125	GABBR1	Gamma-aminobutyric acid (GABA) B receptor, 1	2550
126	GABRA3	Gamma-aminobutyric acid (GABA) A receptor, alpha 3	2556
127	GAS5	Growth arrest-specific 5 (non-protein coding)	60674
128	GATM	Glycine amidinotransferase	2628
129	GBP1	Guanylate binding protein 1, interferon-inducible	2633
130	GJD3	Gap junction protein, delta 3, 31.9kDa	125111
131	GLP1R	Glucagon-like peptide 1 receptor	2740
132	GLRX3	Glutaredoxin 3	10539
133	GP2	Glycoprotein 2 (zymogen granule membrane)	2813
134	GPNMB	Glycoprotein (transmembrane) nmb	10457
135	GPR126	G protein-coupled receptor 126	57211
136	GPR137B	G protein-coupled receptor 137B	7107
137	GPRC6A	G protein-coupled receptor, family C, group 6, member A	222545
138	GSN	Gelsolin	2934
139	GSTCD	Glutathione S-transferase, C-terminal domain containing	79807
140	GTF3C3	General transcription factor IIIC, polypeptide 3, 102kDa	9330
141	H2AFX	H2A histone family, member X	3014
142	HAMP	Hepcidin antimicrobial peptide	57817
143	HDAC4	Histone deacetylase 4	9759
144	HERC2	HECT and RLD domain containing E3 ubiquitin protein ligase 2	8924
145	HHIP	Hedgehog interacting protein	64399
146	HIST1H2BK	Histone cluster 1, H2bk	85236
147	HJURP	Holliday junction recognition protein	55355
148	HLA-A	Major histocompatibility complex, class I, A	3105
149	HLA-DRA	Major histocompatibility complex, class II, DR alpha	3122
150	HLA-DRB5	Major histocompatibility complex, class II, DR beta 5	3127
151	HP	Haptoglobin	3240
152	HTR4	5-hydroxytryptamine (serotonin) receptor 4, G protein-coupled	3360
153	IFI44	Interferon-induced protein 44	10561
154	IFNG	Interferon, gamma	3458
155	IGFBP6	Insulin-like growth factor binding protein 6	3489
156	IGSF10	Immunoglobulin superfamily, member 10	285313
157	IL10	Interleukin 10	3586
158	IL12B	Interleukin 12B	3593
159	IL13	Interleukin 13	3596
160	IL18R1	Interleukin 18 receptor 1	8809
161	IL1A	Interleukin 1, alpha	3552
162	IL1B	Interleukin 1, beta	3553
163	IL23	Interleukin 23, alpha subunit p19	51561

164	IL33	Interleukin 33	90865
165	IL4	Interleukin 4	3565
166	IL6	Interleukin 6 (interferon, beta 2)	3569
167	INCENP	Inner centromere protein antigens 135/155kda	3619
168	INMT	Indolethylamine N-methyltransferase	11185
169	IRF1	Interferon regulatory factor 1	3659
170	ITGAX	Integrin, alpha X (complement component 3 receptor 4 subunit)	3687
171	ITGB6	Integrin, beta 6	3694
172	KCNE2	Potassium voltage-gated channel, Isk-related family, member 2	9992
173	KCNJ15	Potassium inwardly-rectifying channel, subfamily J, member 15	3772
174	KHNYN	KH and NYN domain containing	23351
175	KIF1A	Kinesin family member 1A	547
176	KIT	V-kit sarcoma viral oncogene homolog	3815
177	KLHL6	Kelch-like 6 (Drosophila)	89857
178	KLK1	Kallikrein 1	3816
179	KPNA6	Karyopherin alpha 6 (importin alpha 7)	23633
180	KRT79	Keratin 79	338785
181	LAMP3	Lysosomal-associated membrane protein 3	27074
182	LAPTM5	Lysosomal protein transmembrane 5	7805
183	LBP	Lipopolysaccharide binding protein	3929
184	LECT1	Leukocyte cell derived chemotaxin 1	11061
185	LEPR	Leptin receptor	3953
186	LILRA5	Leukocyte immunoglobulin-like receptor,subfamily A,member 5	353514
187	LILRB4	Leukocyte immunoglobulin-like receptor,subfamily B,member 4	11006
188	LMO7	LIM domain 7	4008
189	LPP	LIM domain containing preferred translocation partner in lipoma	4026
190	LRP1	Low density lipoprotein receptor-related protein 1	4035
191	LRRC33	Leucine rich repeat containing 33	375387
192	LRRC6	Leucine rich repeat containing 6	23639
193	LUM	Lumican	4060
194	LY75	Lymphocyte antigen 75	4065
195	LYVE1	Lymphatic vessel endothelial hyaluronan receptor 1	10894
196	MAP3K6	Mitogen-activated protein kinase kinase kinase 6	9064
197	MC5R	Melanocortin 5 receptor	4161
198	MCEMP1	Mast cell expressed membrane protein 1	199675
199	MCL1	Myeloid cell leukemia sequence 1 (BCL2-related)	4170
200	MCRS1	Microspherule protein 1	10445
201	MECOM	MDS1 and EVI1 complex locus	2122
202	MEX3A	Mex-3 homolog A (C. Elegans)	92312
203	MFAP2	Microfibrillar-associated protein 2	4237

204	MLC1	Megalencephalic leukoencephalopathy with subcortical cysts 1	23209
205	MMP15	Matrix metallopeptidase 15 (membrane-inserted)	4324
206	MMP3	Matrix metallopeptidase 3 (stromelysin 1, progelatinase)	4314
207	MPEG1	Macrophage expressed 1	219972
208	MRC1	Mannose receptor, C type 1	4360
209	MRC2	Mannose receptor, C type 2	9902
210	MRPL21	Mitochondrial ribosomal protein L21	219927
211	MRPS17	Mitochondrial ribosomal protein S17	51373
212	MSLN	Mesothelin	10232
213	MSR1	Macrophage scavenger receptor 1	4481
214	MX2	Myxovirus (influenza virus) resistance 2 (mouse)	4600
215	MYBPC1	Myosin binding protein C, slow type	4604
216	NAAA	N-acylethanolamine acid amidase	27163
217	NCR3	Natural cytotoxicity triggering receptor 3	259197
218	NDNF	Neuron-derived neurotrophic factor	79625
219	NEAT1	Nuclear paraspeckle assembly transcript 1	283131
220	NEB	Nebulin	4703
221	NELL1	NEL-like 1 (chicken)	4745
222	NETO2	Neuropilin (NRP) and tolloid (TLL)-like 2	81831
223	NHSL2	NHS-like 2	340527
224	NME7	NME/NM23 family member 7	29922
225	NPPA	Natriuretic peptide A	4878
226	NR1D2	Nuclear receptor subfamily 1, group D, member 2	9975
227	NRROS	Negative regulator of reactive oxygen species	375387
228	OAS2	2'-5'-oligoadenylate synthetase 2, 69/71kDa	4939
229	OLR1	Oxidized low density lipoprotein (lectin-like) receptor 1	4973
230	OSMR	Oncostatin M receptor	9180
231	OVOS2	Ovostatin 2	144203
232	PAPSS2	3'-phosphoadenosine 5'-phosphosulfate synthase 2	9060
233	PDE4A	Phosphodiesterase 4A, cAMP-specific	5141
234	PDLIM3	PDZ and LIM domain 3	27295
235	PEG10	Paternally expressed 10	23089
236	PER3	Period homolog 3 (Drosophila)	8863
237	PID1	Phosphotyrosine interaction domain containing 1	55022
238	PLAT	Plasminogen activator, tissue	5327
239	PLET1	Placenta expressed transcript 1	349633
240	PLPPR5	Phospholipid phosphatase related 5	163404
241	PON3	Paraoxonase 3	5446
242	POP4	Processing of precursor 4, ribonuclease P/MRP subunit	10775
243	PPARG	Peroxisome proliferator-activated receptor gamma	5468
244	PPIC	Peptidylprolyl isomerase C (cyclophilin C)	5480
245	PPP1R21	Protein phosphatase 1, regulatory subunit 21	129285
246	PPP1R3A	Protein phosphatase 1, regulatory subunit 3A	5506

247	PPT2	Palmitoyl-protein thioesterase 2	9374
248	PRELP	Proline/arginine-rich end leucine-rich repeat protein	5549
249	PRG4	Proteoglycan 4	10216
250	PRKAR2A	Protein kinase, camp-dependent, regulatory, type II, alpha	5576
251	PRKG2	Protein kinase, cgmp-dependent, type II	5593
252	PSCA	Prostate stem cell antigen	8000
253	PTCH1	Patched 1	5727
254	PTGR1	Prostaglandin reductase 1	22949
255	RAD54L2	RAD54-like 2 (S. Cerevisiae)	23132
256	RAD9B	RAD9 homolog B (S. pombe)	144715
257	RARB	Retinoic acid receptor, beta	5915
258	RCAN2	Regulator of calcineurin 2	10231
259	REG3G	Regenerating islet-derived 3 gamma	130120
260	RETNLB	Resistin like beta	84666
261	RGS18	Regulator of G-protein signaling 18	64407
262	RGS4	Regulator of G-protein signaling 4	5999
263	ROS1	C-ros oncogene 1 , receptor tyrosine kinase	6098
264	RPL28	Ribosomal protein L28	6158
265	RSPH1	Radial spoke head 1 homolog (Chlamydomonas)	89765
266	S100A6	S100 calcium binding protein A6	6277
267	SAMD9L	Sterile alpha motif domain containing 9-like	219285
268	SCARB1	Scavenger receptor class B, member 1	949
269	SCEL	Sciellin	8796
270	SCGB1A1	Secretoglobin, family 1A, member 1 (uteroglobin)	7356
271	SCGB1C1	Secretoglobin, family 1C, member 1	147199
272	SCN3A	Sodium channel, voltage-gated, type III, alpha subunit	6328
273	SCN7A	Sodium channel, voltage-gated, type VII, alpha subunit	6332
274	SCNN1A	Sodium channel, non-voltage-gated 1 alpha subunit	6337
275	SCNN1G	Sodium channel, non-voltage-gated 1, gamma subunit	6340
276	SEC14L3	SEC14-like 3 (S. cerevisiae)	266629
277	SEMA3E	Sema domain, immunoglobulin domain (Ig),<br>short basic domain, secreted, (semaphorin) 3E	9723
278	SEMA7A	Semaphorin 7A, GPI membrane anchor	8482
279	SERPINE1	Serpin peptidase inhibitor, clade E (nexin, plasminogen activator inhibitor type 1), member 1	5054
280	SETD5	SET domain containing 5	55209
281	SFMBT2	Scm-like with four mbt domains 2	57713
282	SFTA2	Surfactant associated 2	389376
283	SFTPA1	Surfactant protein A1	653509
284	SFTPC	Surfactant protein C	6440
285	SLC13A4	Solute carrier family 13 (sodium/sulfate symporters), member 4	26266
286	SLC16A7	Solute carrier family 16, member 7	9194
287	SLC1A4	Solute carrier family 1, member 4	6509

288	SLC22A24/ NET46	Solute carrier family 22, member 24	283238
289	SLC24A4	Solute carrier family 24 (sodium/potassium/calcium exchanger), member 4	123041
290	SLC34A2	Solute carrier family 34 (sodium phosphate), member 2	10568
291	SLC36A2	Solute carrier family 36 (proton/amino acid symporter), member 2	153201
292	SLC39A8	Solute carrier family 39 (zinc transporter), member 8	64116
293	SLFN5	Schlafen family member 5	162394
294	SLFN12L	Schlafen family member 12 like	100506736
295	SLURP1	Secreted LY6/PLAUR domain containing 1	57152
296	SNX10	Sorting nexin 10	29887
297	SOD3	Superoxide dismutase 3, extracellular	6649
298	SPATA9	Spermatogenesis associated 9	83890
299	SPCS3	Signal peptidase complex subunit 3 homolog (S. Cerevisiae)	60559
300	SPEN	Spen homolog, transcriptional regulator (Drosophila)	23013
301	SPINK5	Serine peptidase inhibitor, Kazal type 5	11005
302	SPP1	Secreted phosphoprotein 1	6696
303	SREK1	Splicing regulatory glutamine/lysine-rich protein 1	140890
304	ST6GALNAC2	ST6 (alpha-N-acetyl-neuraminyl-2,3-beta-galactosyl-1,3) -N-acetylgalactosaminide alpha-2,6-sialyltransferase 2	10610
305	STBD1	Starch binding domain 1	8987
306	STMN2	Stathmin-like 2	11075
307	STRA13	Stimulated by retinoic acid 13 homolog (mouse)	201254
308	STX8	Syntaxin 8	9482
309	SYCE1	Synaptonemal complex central element protein 1	93426
310	SYCP3	Synaptonemal complex protein 3	50511
311	TC2N	Tandem C2 domains, nuclear	123036
312	TCF20	Transcription factor 20 (AR1)	6942
313	TECRL	Trans-2,3-enoyl-coa reductase-like	253017
314	TF	Transferrin	7018
315	TFF2	Trefoil factor 2	7032
316	TGFB2	Transforming growth factor, beta 2	7042
317	TGM2	Transglutaminase 2	7052
318	THSD4	Thrombospondin, type I, domain containing 4	79875
319	TIFAB	TRAF-interacting protein with forkhead-associated domain, family member B	497189
320	TINAG	Tubulointerstitial nephritis antigen	27283
321	TLE3	Transducin-like enhancer of split 3 (E(sp1) homolog, Drosophila)	7090
322	TLR2	Toll-like receptor 2	7097
323	TLR8	Toll-like receptor 8	51311
324	TM4SF1	Transmembrane 4 L six family member 1	4071
325	TMEM139	Transmembrane protein 139	135932
326	TMEM178A	Transmembrane protein 178A	130733

327	TMEM181	Transmembrane protein 181	57583
328	TMEM2	Transmembrane protein 2	23670
329	TMSB4X	Thymosin beta 4, X-linked	7114
330	TNF	Tumor necrosis factor	7124
331	TNFRSF1B	TNF receptor superfamily member 1B	7133
332	TNNC2	Troponin C type 2 (fast)	7125
333	TNNT3	Troponin T type 3 (skeletal, fast)	7140
334	TNS1	Tensin 1	7145
335	TRABD2B	TraB domain containing 2B	388630
336	TRIM56	Tripartite motif containing 56	81844
337	TRRAP	Transformation/transcription domain-associated protein	8295
338	TTC5	Tetratricopeptide repeat domain 5	91875
339	TYROBP	TYRO protein tyrosine kinase binding protein	7305
340	UACA	Uveal autoantigen with coiled-coil domains and ankyrin repeats	55075
341	UBAP2L	Ubiquitin associated protein 2-like	9898
342	UBR4	Ubiquitin protein ligase E3 component n-recognin 4	23352
343	UGGT2	UDP-glucose glycoprotein glucosyltransferase 2	55757
344	UPK1B	Uroplakin 1B	7348
345	VCAM1	Vascular cell adhesion molecule 1	7412
346	VEGFC	Vascular endothelial growth factor C	7424
347	VEPH1	Ventricular zone expressed PH domain homolog 1 (zebrafish)	79674
348	VPREB3	Pre-B lymphocyte 3	29802
349	VPS28	Vacuolar protein sorting 28 homolog (S. Cerevisiae)	51160
350	WDR45	WD repeat domain 45	11152
351	WIF1	WNT inhibitory factor 1	11197
352	XDH	Xanthine dehydrogenase	7498
353	ZKSCAN3	Zinc finger with KRAB and SCAN domains 3	80317
354	ZXDB	Zinc finger, X-linked, duplicated B	158586
355	ZZEF1	Zinc finger, ZZ-type with EF-hand domain 1	23140

Supplementary table ST2: Summary of the transcript (Gene Paint; mouse embryo) and protein (Human Protein Atlas) expression domains of the significantly associated chronic obstructive pulmonary disease (COPD) genes.

**Protein**	**Gene Paint** [Mouse embryonic (E) lung]	**Staining intensities in Human Lung (Human Protein Atlas)**			
	**Pseudoglandular stage (E14.5)**	**Human Lung Macrophages**	**Human Lung Pneumocytes**	**Human Nasopharynx (Respiratory epithelial cells)**	**Human Bronchus (Respiratory epithelial cells)**
**ABCA10 (ABCA8A)**	Not detectable	Medium	Not detectable	Medium	High
**ADAM19**	Detectable	Not detectable	Not detectable	Low	Not detectable
**BHLHE41**	Not detectable	Not detectable	Not detectable	Not detectable	Not detectable
**CD200**	Detectable	Medium	High	Medium	Medium
**CYBB**	Detectable	Not detectable	Not detectable	Not detectable	Low
**GATM**	Not detectable	Not detectable	Not detectable	Not detectable	Not detectable
**GBP1**	Not detectable	Low	Not detectable	Not detectable	Not detectable
**HJURP (MFLEG1)**	Detectable	Medium	Low	Medium	Medium
**KIT**	Detectable	Medium	Not detectable	Not detectable	Not detectable
**LEPR**	Detectable	Medium	Not detectable	Medium	Medium
**LMO7**	Detectable	Not detectable	High	High	High
**LRP1**	Detectable	Medium	Low	Low	Not detectable
**MCRS1**	Detectable	Medium	High	High	Medium
**POP4**	Detectable	Medium	Not detectable	Medium	Medium
**PTCH1**	Detectable	Medium	Medium	High	Medium
**SCN7A**	Not detectable	High	High	Medium	Medium
**SLFN12L**	Not detectable	Medium	Low	Low	Medium
**TLR8**	Not detectable	High	Not detectable	Low	Low
**TTC5**	Not detectable	Medium	Low	Medium	Medium
**VEPH1**	Not detectable	Medium	Low	Medium	Medium

Supplementary table ST3: Analysis of lung transcript expression of the associated 20 genes in chronic obstructive pulmonary disease (COPD) and/ or emphysematous lung tissues using available datasets [GSE: 29133, 22148, 1650, 47460 and 54837] in Genome Expression Omnibus (GEO) database. ↓**: Decreased** ↑**: Increased** ✓**: significantly altered**

**Associated Genes**	**COPD alveolar epithelial type-II cells GSE: 29133 Fujino et al., 2012**	**COPD stage 2 to 4 compared among each other GSE: 22148 Dave et al., 2011**			**Smokers with severe emphysema GSE: 1650 Spira et al., 2004**	**4 stages of COPD compared to controls COPD GSE: 47460 Peng et al., 2016**				**Stage 2 to 4 compared to stage 1 COPD GSE: 54837 Singh et al., 2014**		
	**Case compared to control**	**Stage 3 vs stage 2**	**Stage 4 vs stage 2**	**Stage 4 vs stage 3**	**Emphysema vs Non-emphysematous lung tissue**	**Stage 1 vs control**	**Stage 2 vs control**	**Stage 3 vs control**	**Stage 4 vs control**	**Stage 2 vs stage 1**	**Stage 3 vs stage 1**	**Stage 4 vs stage 1**
ABCA10									↓✓			
ADAM19									↑✓			
BHLHE41	↓✓	↓✓	↓✓			↑✓	↑✓				↓✓	
CD200		↑✓										
CYBB	↑✓	↓✓	↓✓			↑✓	↑✓		↑✓	↑✓	↑✓	↑✓
GATM		↓✓			↑✓	↑✓						↑✓
GBP1	↑✓									↑✓	↑✓	
HJURP			↓✓									
KIT						↑✓						
LEPR							↓✓		↓✓			
LMO7		↓✓	↓✓								↓✓	↓✓
LRP1		↓✓	↓✓		↓✓		↑✓				↑✓	↑✓
MCRS1		↓✓										
POP4		↓✓	↓✓							↑✓		
PTCH1		↓✓	↓✓	↓✓							↓✓	
SCN7A		↑✓										
SLFN12L												
TLR8						↑✓	↑✓					↑✓
TTC5											↓✓	
VEPH1				↓✓				↓✓			↑✓	

Supplementary table ST4: Analysis of l transcript expression of the associated 20 genes in mouse cigarette smoke exposed lungs using available datasets [GSE: 8790, 7310, 17737, and 6205] in Genome Expression Omnibus (GEO) database. **↓: Decreased ↑: Increased ✓: significantly altered**

**Associated Genes**	**Cigarette smoke (CS) induced emphysema GSE: 8790 Rangaswamy et al., 2009 (CS exposed vs control)**			**Cigarette smoke exposed neonatal mice GSE: 7310 McGrath-Morrow et al., 2007**	**Nose-only exposure of 4% mainstream cigarette smoke GSE: 17737 Contribution by Hu G and Podolin P https://www.ncbi.nlm.nih.gov/geo/query/acc.cgi?acc=GSE17737 (CS exposed vs control)**			**Gene expression analysis using lung transcriptomes in response to cigarette smoke- GSE:76205 Miller et al., 2017 (CS exposed vs control)**					
	**8 days**	**1.5 months**	**6 months**	**CS exposed vs control**	**2 weeks**	**12 weeks CS**	**24 weeks**	**1 day**	**7 day**	**1 month**	**3 months**	**6 months**	**9 months**
Abca8a (Human ortholog ABCA10)		↓ ✓				↓✓	↓✓						
Adam19				↑✓		↑✓	↑✓						
Bhlhe41					↑✓				↑✓	↑✓	↑✓		↑✓
Cd200	↓✓	↓✓									↑✓		
Cybb					↑✓	↑✓	↑✓						
Gatm				↓✓	↑✓	↑✓	↑✓						
Gbp1													
Hjurp			↓✓			↓✓							
Kit	↓✓				↑✓								
Lepr	↓✓			↑✓	↑✓	↓✓	↓✓				↑✓		
Lmo7	↓✓						↓✓				↑✓		
Lrp1					↑✓								
Mcrs1													
Pop4							↑✓						
Ptch1													
Scn7a	↓✓	↑✓	↓✓			↓✓	↓✓				↑✓		
Slfn12l													
Tlr8							↑✓		↑✓				
Ttc5													
Veph1											↑✓		

Supplementary table ST5: The difference in minor allele frequencies of the associated single nucleotide polymorphisms (SNPs) between Korean population and global population indicates the influence of ethnicity on the findings. The Korean population data was accessed from the KoreanDB: http://152.99.75.168/KRGDB/menuPages/firstInfo.jsp and http://152.99.75.168/KRGDB/browser/mainBrowser.jsp
Global SNP data(dbSNP database): https://www.ncbi.nlm.nih.gov/SNP/

**Gene**	**Associated SNP**	**Korean DB Alt Allele frequency (662 samples)**	**Korean DB Alt Allele frequency (1100 samples)**	**dbSNP Global Alt Allele frequency/sample size**
ABCA10	rs4968849	0.42	0.42	A=0.2887/1446
ADAM19	rs1422795	0.14	0.17	C=0.4145/2076
BHLHE41	rs11048413	0.2	0.1	A=0.3706/1856
CD200	rs1131199	0.7	0.76	C=0.3397/1701
GATM	rs1288775	0.85	0.84	T=0.3814/1910
GBP1	rs1048425	0.54	0.53	G=0.2817/1411
HJURP	rs2286430	0.39	0.4	T=0.3460/1733
KIT	rs3822214	0.03	0.03	C=0.0645/323
LEPR	rs1137101	0.86	0.83	A=0.4157/2082
SCN7A	rs7565062	0.67	0.67	G=0.3113/1559
SCN7A	rs6738031	0.66	0.66	C=0.2554/1279
SLFN12L	rs2304968	0.72	0.7	T=0.2452/1228
TLR8	rs3764880	0.82	0.75	G=0.4638/1751
TTC5	rs3742945	0.83	0.82	T=0.2544/1274
VEPH1	rs11918974	0.38	0.39	G=0.2506/1255

Supplementary Figure S1: Analysis of protein domain and functional sites in the "A Disintegrin and metallopeptidase domain 19" (ADAM19)

ADAM19 (A Disintegrin And Metallopeptidase domain 19)

SNP	Genomic location	Ref amino acid-Position-Alt aminoacid	Significance	Amino acid Properties	
				Serine	Glycine
rs1422795	Chr5: T-156936364-C	S-17-G	P = 0.0004	Polar	Nonpolar
				Neutral	Neutral

Protein domains and functional sites

ADAM19 (Wild Type)

Upper case represents match positions, lower case insert positions, and the '-' symbol represents deletions relative to the matching profile.

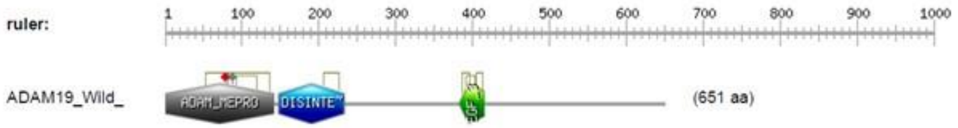

PS50215 **ADAM_MEPRO** *ADAM type metalloprotease domain profile :*

1 - 141: **score =** 27.751

```
----------------------------------------------------------MC
EVSENPYSTLWSFLSWRRKLL-AQKYHDNAQLITGMSFHGTTIGLAPLMAMCSVYQSGGV
NMDHSENAIGVAATMAHEMGHNFGMTHDSaDCCSASAadGGCIMAAATGHPFPKVFNGCN
RRELDRYLQSGGGMCLSNMP
```

ADAM19 (Variant induced)

Upper case represents match positions, lower case insert positions, and the '-' symbol represents deletions relative to the matching profile.

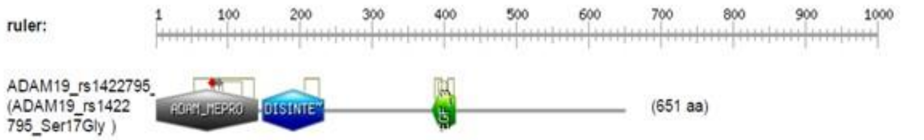

PS50215 **ADAM_MEPRO** *ADAM type metalloprotease domain profile :*

1 - 141: **score =** 27.583

```
----------------------------------------------------------MC
EVSENPYSTLWSFLGWRRKLL-AQKYHDNAQLITGMSFHGTTIGLAPLMAMCSVYQSGGV
NMDHSENAIGVAATMAHEMGHNFGMTHDSaDCCSASAadGGCIMAAATGHPFPKVFNGCN
RRELDRYLQSGGGMCLSNMP
```

Supplementary Figure S2: Transcript (Gene Paint; mouse embryo) and protein expression (Human Protein atlas; normal lung) domain of holliday junction recognition protein (*HJURP*)

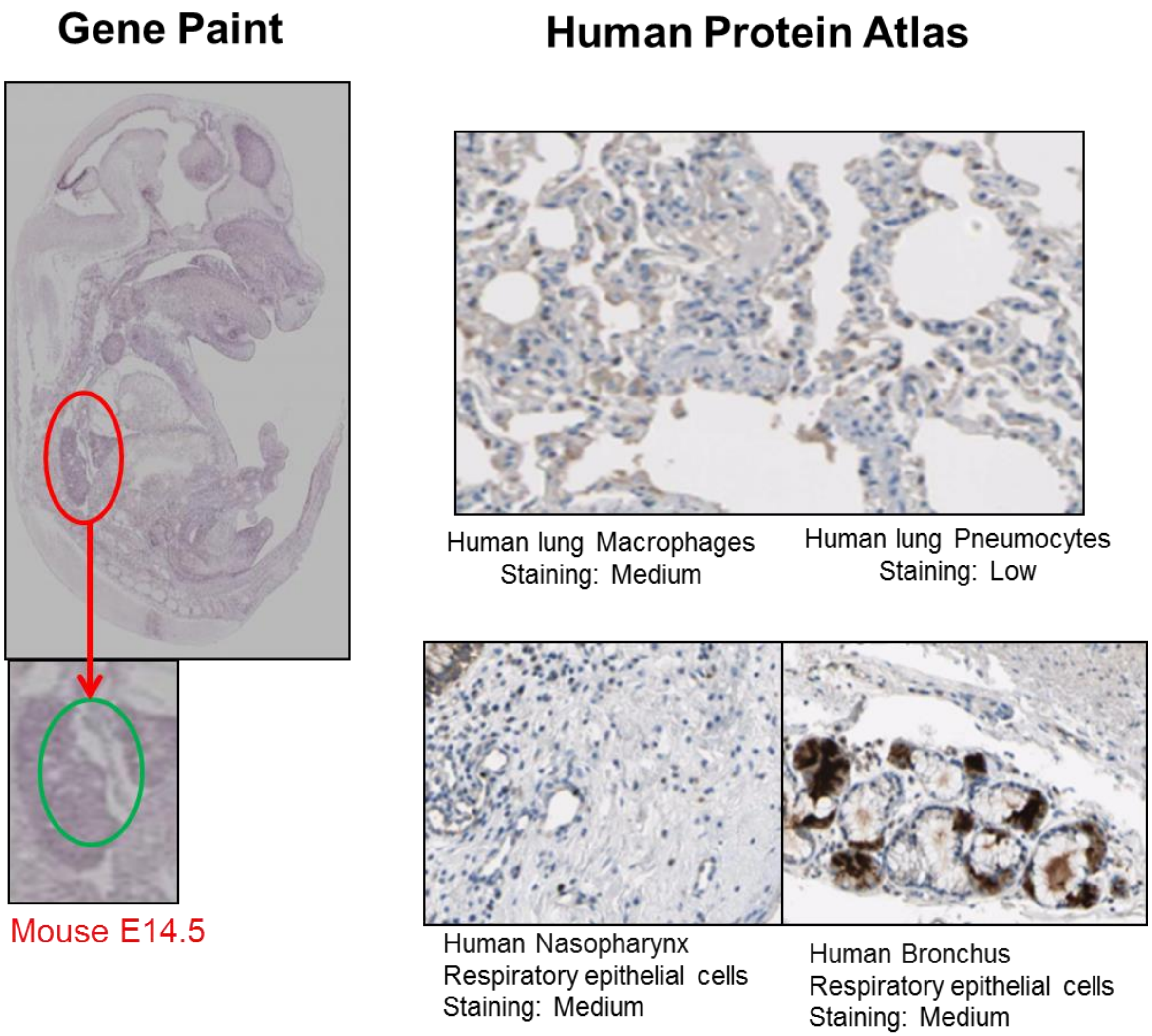

Supplementary Figure S3: Transcript (Gene Paint; mouse embryo) and protein expression (Human Protein atlas; normal lung) domain of microspherule protein 1 (MCRS1).

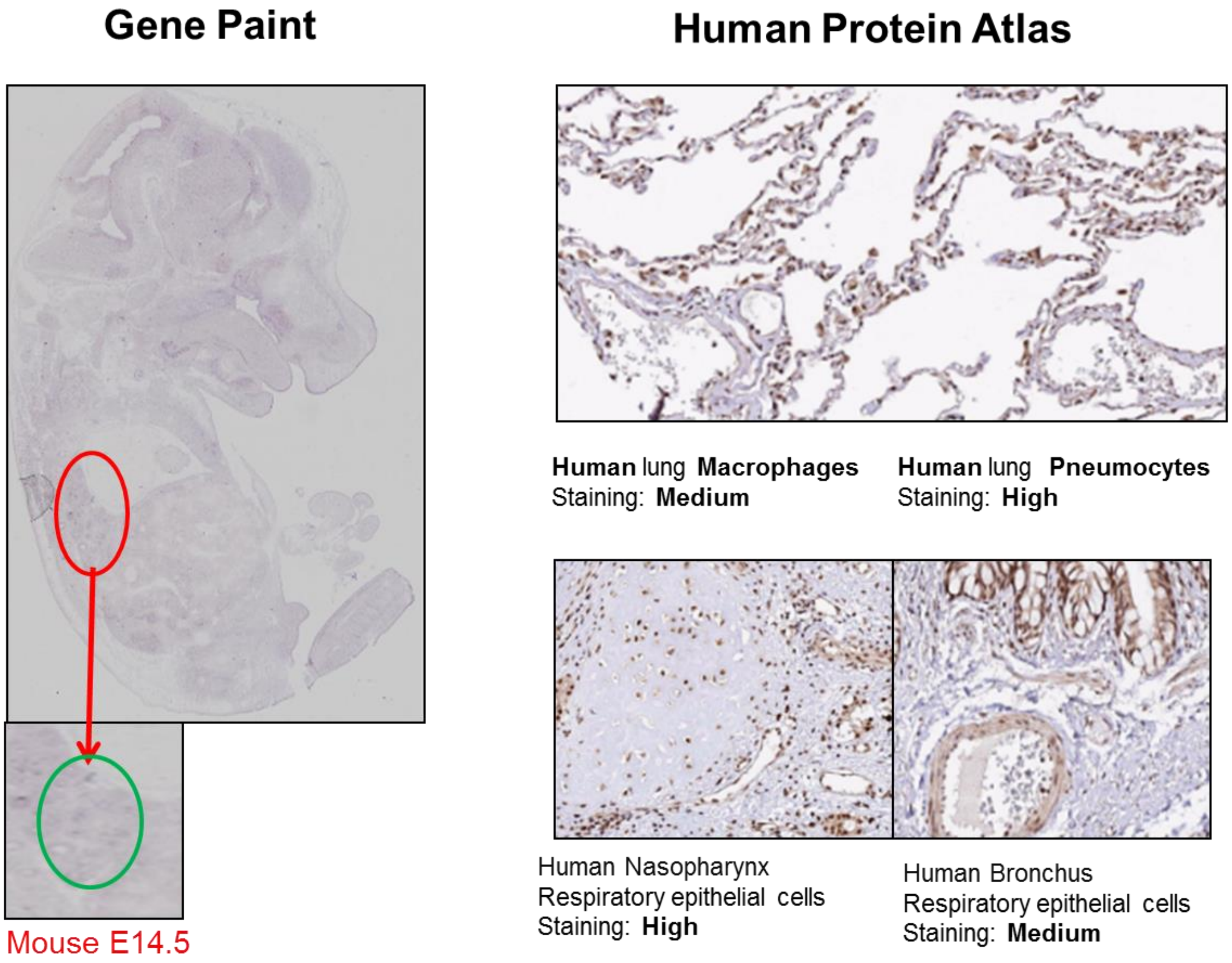

Supplementary Figure S4: Protein expression (Human Protein atlas; normal lung) domain of toll like receptor 8 (TLR8).

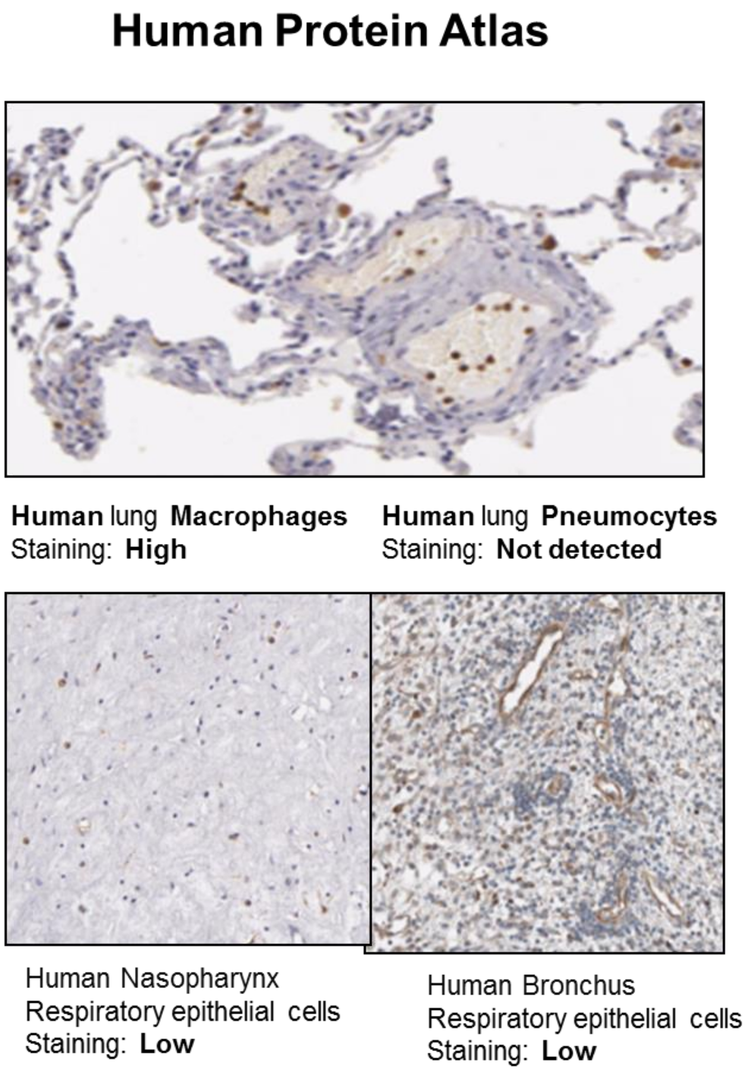

Supplementary Figure S5: Gene-set enrichment analysis for the associated 20 genes for (A) cellular component enrichment (B) biological process enrichment (C) molecular function enrichment (D) diseases enrichment using Enrichr interactive enrichment analysis tool (Chen et al., 2013.).

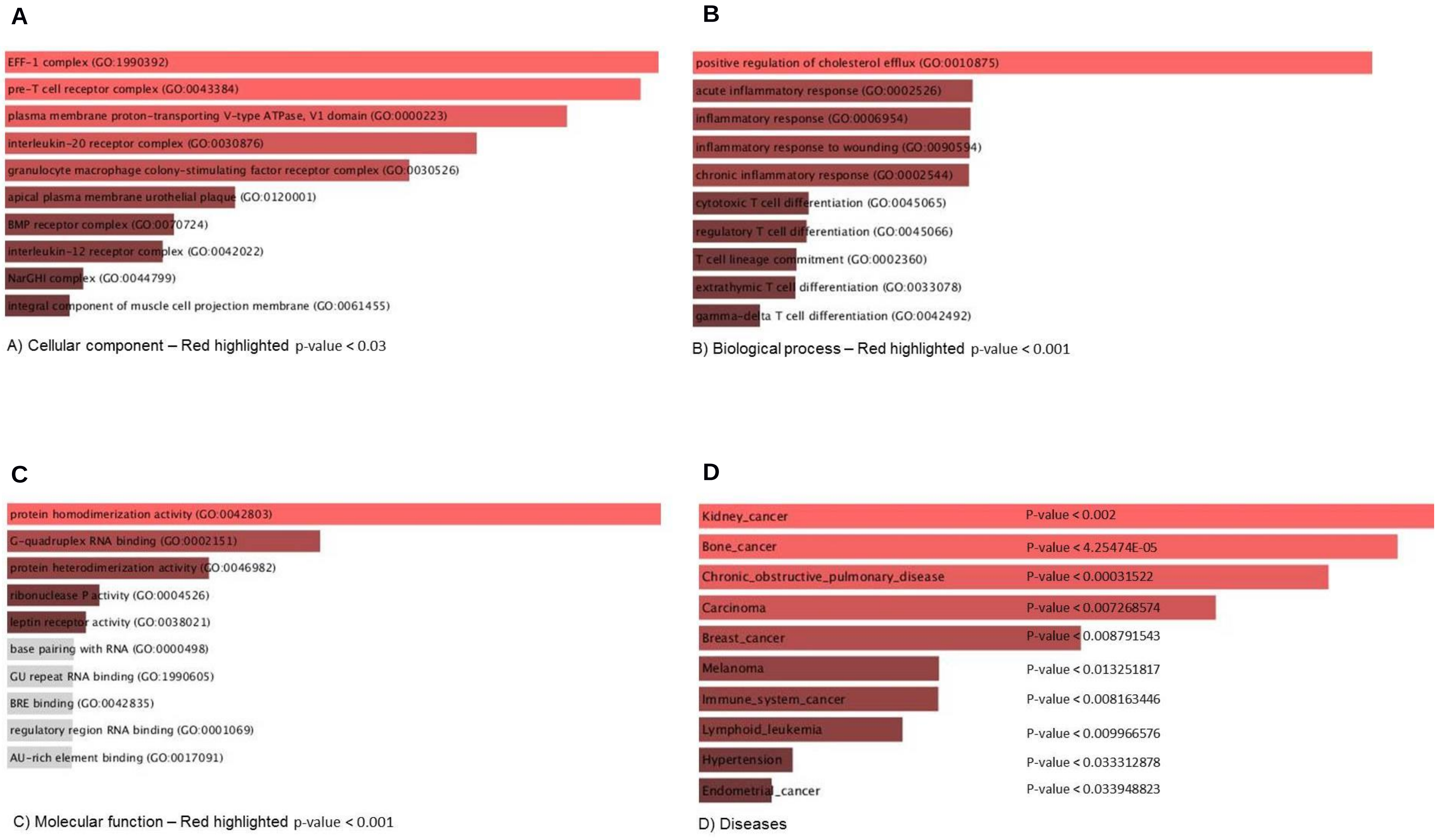

Supplement: Supplementary file 1 — Table S1. List of the genes screened for association to higher Chronic Obstructive Pulmonary Disease (COPD) susceptibility. Table S2. Summary of the transcript (Gene Paint; mouse embryo) and protein (Human Protein Atlas) expression domains of the significantly associated chronic obstructive pulmonary disease (COPD) genes. Table S3. Analysis of lung transcript expression of the associated 20 genes in chronic obstructive pulmonary disease (COPD) and/ or emphysematous lung tissues using available datasets [GSE: 29133, 22,148, 1650, 47,460 and 54,837] in Genome Expression Omnibus (GEO) database. ↓: Decreased ↑: Increased ✓: significantly altered. Table S4. Analysis of l transcript expression of the associated 20 genes in mouse cigarette smoke exposed lungs using available datasets [GSE: 8790, 7310, 17,737, and 6205] in Genome Expression Omnibus (GEO) database. ↓: Decreased ↑: Increased ✓: significantly altered. Table S5. The difference in minor allele frequencies of the associated single nucleotide polymorphisms (SNPs) between Korean population and global population indicates the influence of ethnicity on the findings. The Korean population data was accessed from the KoreanDB: http://152.99.75.168/KRGDB/menuPages/firstInfo.jsp and http://152.99.75.168/KRGDB/browser/mainBrowser.jsp Global SNP data(dbSNP database): https://www.ncbi.nlm.nih.gov/SNP/. Figure S1. Analysis of protein domain and functional sites in the “A Disintegrin and metallopeptidase domain 19” (ADAM19). Figure S2. Transcript (Gene Paint; mouse embryo) and protein expression (Human Protein atlas; normal lung) domain of holliday junction recognition protein (HJURP). Figure S3. Transcript (Gene Paint; mouse embryo) and protein expression (Human Protein atlas; normal lung) domain of microspherule protein 1 (MCRS1). Figure S4. Protein expression (Human Protein atlas; normal lung) domain of toll like receptor 8 (TLR8). Figure S5. Gene-set enrichment analysis for the associated 20 genes for (A) cellular componen [file 12931_2018_795_MOESM1_ESM.pdf]
